# Public Health Impact of Legal Termination of Pregnancy in the US: 40 Years Later

**DOI:** 10.6064/2012/980812

**Published:** 2012-12-13

**Authors:** John M. Thorp

**Affiliations:** Department of Obstetrics and Gynecology, School of Medicine, University of North Carolina, Chapel Hill, NC 27599, USA

## Abstract

During the 40 years since the US Supreme Court decision in Doe versus Wade and Doe versus Bolton, restrictions on termination of pregnancy (TOP) were overturned nationwide. The use of TOP was much wider than predicted and a substantial fraction of reproductive age women in the U.S. have had one or more TOPs and that widespread uptake makes the downstream impact of any possible harms have broad public health implications. While short-term harms do not appear to be excessive, from a public perspective longer term harm is conceiving, and clearly more study of particular relevance concerns the associations of TOP with subsequent preterm birth and mental health problems. Clearly more research is needed to quantify the magnitude of risk and accurately inform women with the crisis of unintended pregnancy considering TOP. The current US data-gathering mechanisms are inadequate for this important task.

## 1. Introduction

This is a paper written on the 40th anniversary of the US Supreme Court decisions in *Roe *versus* Wade *and *Doe *versus* Bolton*, in which termination of pregnancy (TOP) restrictions were overturned in all fifty states [1]. This profound sociocultural shift in paradigm and practice has had substantial impact on the lives of women and families in the US and around the world. I am writing from the perspective of an obstetrics and gynecology clinician who has witnessed much of this change during his career. A similar approach from a personal perspective has been used by others on earlier anniversaries [2–7].

 Rather than the traditional recitation of limitations at the end of the paper, I have chosen to inform the readers of those *a priori*. Thus, it can process the evidence cited and conclusions made with those caveats in mind. My hope would be that by providing context from the outset, the reader will be able to more thoughtfully consider the material presented.

 First, this is not a systematic review. The relevant literature has not been systematically searched, read by multiple reviewers, graded against a standard, or quantified by meta-analysis. A more subjective and qualitative approach has been chosen. This approach was selected due to the lack of randomized controlled trials for a procedure that cannot be ethically assigned by chance, the inherent difficulties in synthesizing observational data, and the ethical controversy that swirls underneath this topic. Meta-analyses on appropriate outcomes are included in the paper when they have been done.

 Next, the paper is geographically parochial and limited to the US. This is due to the limitations of the author and the fact that the US experience is often generalized to the rest of the developed world. Articles from other countries are cited for comparison purposes.

 The language used to describe these procedures is fraught with inaccuracy and often overlaps with clinical language used to describe spontaneous pregnancy loss. This is confusing to readers and at times alarming to patients [8]. In this paper the phrase “termination of pregnancy” (abbreviated as TOP) will be used throughout. Use of the abbreviation TOP reflects the editorial policy of the *British Journal of Obstetrics and Gynaecology* and has been forged after numerous discussions among the editors, to which the author has been privy. 

 Finally, and most importantly, the ethical principle of autonomy precludes random assignment to TOP. Thus, randomized controlled trials of TOP exposure versus no exposure are impossible to conduct. By definition there is no “Level One” or “Grade A” evidence about TOP [9]. Given that women who choose TOP differ from nonpregnant women, those who continue a pregnancy, or those who suffer a spontaneous loss, unmeasured residual confounding plagues investigators and readers trying to understand the biologic implications of the procedure. The appropriate nonexposed control is a source of much disagreement. Whether one discerns true benefit or substantial harm from observational data about TOP, conclusions will always be limited along those lines.

## 2. Background

 Before the US Supreme Court decisions mentioned earlier were issued, TOP in the US was severely restricted. With that decision, access to the procedure was greatly expanded and the uptake of TOP procedures was higher than experts predicted. From most recent sources, a prototypical US woman will have two children [10, 11]. Nearly half of all US women will have an unplanned or unintended pregnancy (when intentionality is broadly defined) and somewhere around half of those will elect to undergo TOP. Thus, between menarche and menopause, in the US, one out of three women will undergo TOP. This makes TOP “one of the most common medical interventions” [12–15].

 In the US, approximately 1.2 million TOPs were performed in 2009, while there were 4.5 million live births [13]. Around 90% of TOPs in the US will be done in the first trimester, with the remainder in the second and third trimesters. Around 1-2% of TOPs in the US are done because of serious maternal illness, such as cancer or pulmonary hypertension, or an abnormality detected in the fetus, such as trisomy or neural tube defect [14]. Until the past decade, almost all TOPs in the US were performed surgically. Since 2000, medical TOP has become more widely used, building on experience from Western Europe. Medical TOP involves use of the progesterone antagonist mifepristone and the prostaglandin misoprostol. Around 10% of TOPs in the US are completed medically [16, 17]. 

## 3. Epidemiology

 Unlike numerous other countries, the US does not have a systematic method of keeping track of TOP procedures [18–22]. TOPs are not registered by the government, and there is no mandatory reporting so the true incidence cannot be ascertained. Moreover, TOPs cannot be linked to other sources of health data such as birth or death certificates, thereby making precise calculation of mortality rates or subsequent birth outcomes impossible. This lack of registration of TOP in the US means that observational data from other countries is more reliable in determining short- and long-term health effects. To quote a famous US women's health epidemiologist, the current situation could be described as “garbage in, garbage out” [23]. 

 Two proximate data sources are used in the US by those interested in TOP epidemiology. The estimated number of TOP procedures in the US is obtained from periodic surveys of all identifiable TOP providers by the Guttmacher Institute. Reporting is voluntary and the surveys are done at irregular intervals up to five years apart. The Guttmacher Institute has a clear political agenda and anyone perusing their website would be hard pressed to not describe it as an advocacy group [24, 25]. 

 The second source is the annual “abortion surveillance report” amalgamated by the US Centers for Disease Control (CDC). Beginning in 1969, state health departments have voluntarily provided annual reports on TOP procedures and patients. These data are incomplete due to the wide variability in state requirements for reporting of TOP procedures, the voluntary nature of participation with some states choosing to not do so periodically, marked variation in the information each state obtains, and the lack of specific funding for TOP data accumulation. For instance, the large US State of California has not reported in the past decade. Thus, any report on TOP epidemiology from the US is fraught with numerous assumptions and lack of any clear standardization [26–30].

 The incidence of TOP is usually stated in one of three ways: total number, rate, and ratio. The TOP rate is the number of TOP procedures per 100 women aged 15 to 44 years (so-called reproductive age). The TOP ratio is the number of TOP procedures per 100 pregnancies. Pregnancy in the ratio includes live births and TOPs and it excludes spontaneous pregnancy losses and intrauterine fetal demises. 

 With these caveats in mind, and despite the limitation of TOP epidemiology, the utilization of TOP soared dramatically after the US Supreme Court decision. TOP numbers peaked in 1988 to 1.59 million, with the peak TOP rate in 1990 of 27.4 TOP/100 women aged 15–44 and the peak TOP ratio in 1983 of 30.4 TOP/100 pregnancies. From 1973 to 2008, Guttmacher estimates that 50 million TOPs were performed. In 2008, US women had 1.2 M TOPs with the TOP rate being 19.6 TOP/1000 women aged 15–44 years [24, 25]. 

 Using estimates from the Guttmacher Institute patient survey, US women in their 20s account for 57% of all TOPs. Adolescents under 20 obtain 18% of TOP. As to race, in 2008 among women choosing TOP, 36% were non-Hispanic white, 30% were non-Hispanic black, and 25% were Hispanic. Single women (never married and not cohabitating) had 45% of TOP procedures in that year and 61% of women seeking TOP were mothers to at least one child. In terms of socioeconomic status, 42% of women seeking TOP have incomes below the US poverty level [24, 25]. In the month they conceived, over half of the women seeking TOP were contracepting with the majority using oral contraceptives or condoms. As one might predict, inconsistent use of contraceptives in the preceding month is common—76% of pill users and 49% of couples relying on condoms [29, 30].

 Almost 90% of TOPs are done in the first trimester (12 weeks from the last menstrual period). Somewhere around 1% of TOPs in the US are performed after 20 weeks. These numbers have remained steady over the epoch this paper covers, although there has been a shift toward lower gestational ages with more TOPs done in the first trimester over the 40-year epoch being reviewed [26–30]. This phenomenon has been attributed to the approval in 2000 by the US Food and Drug Administration of mifepristone for medical TOP. Medical TOP regimens become less effective after 9 weeks and many protocols limit their use thereafter. Concurrent with widespread legalization of TOP in the US in 1973 was the introduction of vacuum aspiration (or suction curettage) which widely became the mechanism of choice for surgical TOP. This procedure can be utilized for TOP up until 13 weeks and includes cervical dilation, either chemically or mechanically, and suction to empty the uterine contents. Procedures for second trimester TOP include induction of labor chemically or mechanically, hysterotomy, hysterectomy, or dilation and evacuation (D&E). D&E TOP is the most common technique in the US, accounting for over 95% of the procedures done after the first trimester [31–35]. 

In the early 1970s, most TOP procedures were done in acute care hospitals. Over time the TOP site of service has shifted from the hospital to the outpatient setting. By 2005, only 5% of TOPs were done in the hospital. Medical TOP approaches have allowed for procedures to shift from the medical setting to the home. Even second trimester D&E procedures for TOP can be done in the outpatient setting [36]. Compared to other developed countries, the US TOP rate is substantially higher than Finland, Switzerland, Germany, and the Netherlands where rates range from 7 to 11/1000 women and ascertainment is far better due to standard registries and record linkage capacity. Only Sweden in 2005 had a TOP rate similar to the US at 20 or so TOPs/1000 reproductive-age women. Women in European countries are more likely to undergo medical TOP than are US women [18–21].

 Thus, despite expert prediction that easing TOP restrictions in the US would culminate in TOP rates less than 5/1000, the utilization of TOP has been much higher than anticipated. This phenomenon has only begun to decline in the past decade despite the widespread availability and improved safety of effective contraceptives. One in ten US women will have had a TOP by the age of 20, one in four by 30, and one in three by 45. Each year in the US 2% of women between 15 and 44 years of age have a TOP. This has caused experts to label TOP as the “most common of medical interventions” [28–30]. Beyond ethical and demographic concerns with widespread use of TOP in the US, it has significant epidemiologic implications. Even modest elevations in hazard ratios associated with TOP would have profound biologic effects due to its common use in the US For instance, if the hazard ratio for preterm birth associated with TOP is 1.3, then the population attributable risk would be 3.2% and therefore over one-quarter of preterm births in developed countries would be attributable to TOP [37]. Harms or benefits associated with such a commonly used procedure, even if rather modest, would ripple through a population and have a large impact. Thus, the need for accurate incidence estimates and linkage to other health records is important to the large number of women who will have a TOP, the 75% of those having TOP who will try to have a child after TOP, and to a society interested in the health of women and children.

## 4. Maternal Mortality

 As described earlier in this paper, the US TOP epidemiology is plagued by the lack of a TOP registry and reliance on what is largely survey methodology [38]. Because this system is voluntary, and also due to the inherent reluctance of surgeons to disclose serious complications such as death, underreporting is a major problem [39]. Comparing CDC Abortion Surveillance reports to deaths reported in the popular press demonstrates that, for instance in 1989, four deaths resulting from TOP did not make it into the state's statistics (the CDC reported zero deaths in that particular year) [38]. 

 In contrast to the US, countries with mandatory reporting and the ability to link birth, abortion, and hospital registries show increased rates of mortality above US estimates and increased relative risk of death after TOP when compared to women having a child. Similar findings have been reported in the US from administrative databases, such as publicly-funded TOPs from California [38]. This casts further doubt on contemporary US surveillance systems to generate reliable estimates for TOP-related mortality.

 Unlike the identification and ascertainment of TOP-related deaths in the US, pregnancy-related deaths are systematically sought, identified, and investigated by state maternal mortality commissions. These entities have the ability to confidentially obtain medical records, review clinician notes, and determine if a pregnancy-related death was preventable. The commissions have operated since the early 20th century. Over the past 30 years more and more US jurisdictions allow electronic linking of birth and death certificates. This automated connection process has greatly increased the number of pregnancy-related deaths identified and allowed a more thorough understanding of the epidemiology of childbirth. TOP lacks any formal registry and thus linking TOP with death is not possible [38].

 TOP is a surgical procedure usually completed in minutes, whereas childbirth encompasses the 40 weeks of pregnancy and 6 weeks postpartum. Thus, TOP and its associated major complication, death, is like a single snapshot, whereas pregnancy and pregnancy-related death are like a feature length film. Moreover, any death during or 6 weeks after pregnancy is labeled pregnancy related and categorized as direct or indirect. A woman who underwent TOP in the first trimester, suffered profound depression, and four weeks later committed suicide would not be labeled a TOP-related death even if the TOP was known about. Conversely, a woman delivering at term who had a similar series of events would be labeled a pregnancy-related death. This differential window of attribution makes direct comparisons misleading. 

 One recent study from Chile explored 50 years of registry data from a country that prohibited TOP in 1989. Such restrictive legislation would have been predicted by many women's health epidemiologists and clinicians to greatly increase childbirth-related deaths if TOPs were truly safer. The contrary occurred with maternal mortality from pregnancy and childbirth continuing to fall even after the TOP restrictive law was passed. Increasing education levels of women had the most favorable impact on the falling childbirth-related maternal mortality rates [40]. 

 TOP epidemiologists lump all deaths together across the full spectrum of gestational age despite the well-known fact that TOP morbidity and mortality increase with advancing gestational age. The fact that TOP most often occurs in the first trimester in the US skews the aggregated mortality numbers used in most comparisons toward the time in pregnancy where TOP procedures are relatively safer. The risk of death associated with TOP increases from one death for every one million TOPs at less than 9 weeks to three per one hundred thousand TOPs at 16–20 weeks to 10 per one hundred thousand at 21 or more weeks in the US [27–30].

 Reardon and Coleman [41] just published an article which looked at maternal mortality for an epoch of 25 years using Danish birth and death records. Their cohort consisted of 463,473 women and they used TOP in the first pregnancy as the exposure of interest, controlling for pregnancy outcomes in subsequent gestations. For women having TOP at <12 weeks, cumulative mortality rates were higher from 180 days to 10 years from the index pregnancy. The association between TOP and cumulative mortality was similar but stronger for TOP >12 weeks gestation. The comparison group was women who delivered after 20 weeks gestation. While far from definitely answering the question, the linkage study does cast doubt on the claim that TOP is safer than pregnancy continuation. 

 Another problem inherent in comparing aggregated deaths for TOP and pregnancy is the failure to control for important confounders other than gestational age when the TOP is performed. Women seeking TOP in the US are younger and presumably healthier, although they are more likely to be single and of lower socioeconomic status which have negative health effects. Thus, failure to control for important confounders makes direct comparisons of crude rates even less accurate [42–46]. 

 In terms of lives lost, current TOP epidemiologic approaches assume that the embryo or fetus has a null moral status and that the loss of a potential human being (which is the stated goal of every TOP procedure) should not be considered. This failure to account for the impact of losing a future citizen has had profound demographic consequences in countries with unrestricted access to TOP, such as the US In a horrible twist reminiscent of the eugenics movements of the 20th century, some US states have even lowered barriers to TOP with the stated intent of lowering the number of individuals needing social support or mental health services [47]. These losses are not captured in mortality statistics that solely value the life of the mother.

Despite the inherent absurdity in comparing death rates from TOP to childbirth, such comparisons continue to be done by prominent clinicians and various advocacy groups [48]. Comparisons are inherently biased and those biases may confuse women considering whether to have a TOP or continue their pregnancy. Differences in ascertainment of deaths, duration of susceptibility to mortality, lack of accounting for gestational age, and choice of appropriate comparison group make these comparisons a fool's errand.

## 5. Short-Term Harms

 Like all other elective surgical or medical procedures, TOP carries the inherent risk of bleeding, infection, and damage to other organs in the genitourinary and gastrointestinal tracts. In addition, TOP procedures have unique risks of incomplete emptying of the uterus and obfuscation of the diagnosis of ectopic pregnancy [49]. As mentioned in the mortality section, the likelihood of harm is dependent on gestational age with risk directly proportional to gestational age. One US study estimates a hazard ratio of 1.38 for TOP complication for each week after a TOP procedure is performed with the same being true for TOP-related mortality [50]. 

 Bleeding or hemorrhage (defined as estimated blood loss > 500cc) occurs in up to 1% of TOPs in the first trimester and up to 2.5% of second trimester TOPs. Causes of excessive blood loss include cervical laceration, uterine perforation, atony, and retained pregnancy (products of conception). While atony may respond to uterine massage or administration of drugs that cause the uterus to contract, the other complications will require additional surgery. Rarely blood transfusion and hysterectomy will be needed [51]. Uterine perforation or puncture by the suction cannula or sharp curette during the TOP occurs in 10–15/1000 procedures. Risk factors include increasing gestational age, uterine leiomyomata, and surgeon inexperience. Perforations can damage pelvic vessels, bowel, bladder, and tubes and ovaries. This complication is the most common inciting event for subsequent laparotomy or laparoscopy to inspect the pelvis for organ damage. “Silent” or unrecognized perforation with organ injury can have severe consequences [52].

 Likewise, the cervix (opening to the uterus) can be torn or lacerated during a TOP. The likelihood of cervical trauma can be greatly reduced by cervical preparation with *Laminaria* or pharmacotherapy prior to TOP [53–55]. Up to 3% of second trimester TOP procedures are complicated by cervical trauma [56]. Unrecognized or inadequately repaired tears are one proposed mechanism for the association between TOP and preterm birth in a subsequent pregnancy. The trauma is hypothesized to culminate in cervical insufficiency.

 Failure to empty the uterus completely or recognize an ectopic gestation during TOP is rare event when ultrasound is used before and after TOP procedures. One concern in the proposed scheme to increase access to TOP by having nurses performing the procedure is their lack of familiarity with diagnostic imaging. These TOP complications when not recognized or attended to can have deleterious health outcomes, including death.

 Infection is the most common short-term complication after TOP [51]. It occurs after 1 to 5% of surgical TOPs and is usually polymicrobial in nature [57]. Antibiotic prophylaxis can reduce this risk in surgical TOP. The so-called septic abortion is the most common presentation and usually responds to broad spectrum antibiotics and evacuation of any retained portions of the pregnancy. Rarely, laparotomy will be needed to drain an abscess or remove an infected uterus. There has been a recent cluster of fatal toxic shock after medical TOP caused by *Clostridium* species. The epidemiology of this complication and role of microbiotic prophylaxis remains to be elucidated [56, 57]. When medical and surgical TOP procedures are directly compared, more women in the medical TOP groups will require surgical evacuation and experience more bleeding, while surgical TOP has more traumatic complications [58–60]. 

## 6. Long-Term Harms

 While there are numerous claims made asserting that TOP has no long-term consequences beyond the immediate complications described above, careful readers will be struck by the paucity of data on this topic, particularly from the US Moreover, as mentioned throughout this paper, all data are observational rather than experimental. Case-control studies are particularly prone to differential reporting of TOP exposure and a serious illness such as a new cancer diagnosis may make a case more forthcoming about a TOP than would be a control of this sensitive issue. In the US, lack of TOP registration and thus inability to conduct cohort studies in which there is no linkage to birth certificates or cancer registries makes all investigators reliant on self-reports of TOP exposure [61]. 

 TOP does not appear to be associated with subsequent increased risk of subfertility, spontaneous abortion, or ectopic pregnancy. There has been shown a consistent, modest association between TOP and placenta previa in a subsequent pregnancy. 

 I will not belabor those findings, but turn to three conditions in which the literature is more comprehensive in reporting links between TOP and the health outcome in question. Those will include preterm birth, breast cancer, and mental health problems. Each is an important women's health outcome with lifetime prevalence greater than 10%. Readers should harken back to the discussion about population attributable risk to refresh their memories that even small increases in risk can have profound health consequences given the fact that one out of three US women will undergo TOP in their reproductive years. 

 Preterm birth (PTB) prior to 37 weeks gestation is the most common cause of infant death and disability in the US and complicates more than one in ten births [62]. The etiology remains unclear, and concurrent with the social experiment of abolishing legal restrictions to TOP in the US initiated by the* Roe *versus* Wade* decision, the US has experienced an ever-increasing rate of PTB. Other changes have occurred over that epoch such as increased use of subfertility treatment, which increases the risk of multifetal gestations and preterm birth. Mechanical trauma to the cervix, infection, and scarring of the endometrium are all putative mechanisms for how TOP could increase the likelihood of PTB [63].

 In 2009, Shah and Zao, on behalf of the “Knowledge Synthesis Group of Determinants of Preterm/Low Birth Weight” conducted a rigorous, systematic meta-analysis [37]. The world's literature was independently reviewed and 37 studies were assimilated. One TOP was associated with increased odds of PTB (OR–1.36, 95% CI 1.24–1.50) and more than one TOP with increased odds of PTB (OR–1.93, 95% CI 1.28–2.71). Moderate statistical heterogeneity was identified between studies and the results persisted after adjustment for known confounders. The population attribute risk was 2.7% and thus up to one-third of PTBs in contemporary perinatal practice in the US could be due to TOP [64]. There are over 130 published studies showing an association between TOP and either preterm birth or its surrogate, low birthweight [65–196].

 In one of the larger and most comprehensive studies published to date, Klemetti et al. recently reported that in first-time mothers, TOP was associated with preterm birth, particularly “very early” preterm births at <28 weeks gestation. Using the Finnish Medical Birth and Abortion Registries to link 300,858 records, they not only showed an association between TOP in a first pregnancy and very early preterm birth, but also showed a “dose response” effect with more TOPs increasing the strength of the association. They call for health care professionals and the public to be warned about the risks [197].

 This phenomenon is of great importance in perinatal epidemiology. Most risk factors for PTB, such as race or multifetal gestation, are not modifiable, while TOP is mutable [62, 63]. In my clinical experience, subsequent pregnancy performance is of paramount importance to a woman facing a crisis or unintended pregnancy. Up to 75% of US women opting for TOP will have a subsequent pregnancy, so the importance of the finding from public health, demographic, and informed consent perspectives should not be underestimated. 

 Studies exploring an association between TOP and breast cancer have had mixed results with some showing small increases in risk and others demonstrating no difference between exposed and unexposed women. The single meta-analysis found a summary odds ratio of 1.3 (95% CI 1.2–1.4) [62] while a US National Cancer Institute review found no effect [198], as did the Collaborative Group on Hormonal Factors in Breast Cancer in a reanalysis of 53 studies [199]. In 2002, our research group presented a novel theory based on the Gail Model, which is widely used by clinicians to predict 5-year and lifetime risk of breast cancer, and has been used as the entry criteria for chemoprevention studies. In this model, a term birth and lactation are protective, reducing future risk, and thus we speculated that a woman choosing TOP and foregoing childbirth would experience a loss of protection. This would be of no mere consequence for younger women as the younger a woman is with her first term pregnancy, the greater protective effect she might enjoy. To our knowledge no one has gathered prospective data to test our hypothesis. This link, or lack thereof, remains a field ripe for further exploration [61].

The story is almost identical to breast cancer for TOP and mental health. Individual studies are mixed, albeit numerous, indicating harm [198, 200–300]. One meta-analysis shows a modest increased risk of mood disorders, suicide, and substance abuse in women having TOP [301], and two reviews by national organizations have concluded that the data are limited and no firm conclusions can be drawn [302]. Problems include what will begin to sound like a familiar litany to readers of this paper—the limits of observational data, lack of US registry data, recall bias, and the list goes on [303]. Again, this is an area that merits investigators' attention, as the population attributable risk for the meta-analysis was that up to 10% of mental health problems, in women who experience a disproportionate share of mental health problems, can be linked to TOP.

## 7. Clinicians Providing Top

 In the US after *Roe *versus* Wade* in 1973, there was an increase in the number of TOP providers up until 1982. After 1982, the number has steadily declined and by 2005 the total was close to the number in 1973. Coincidental with this decline has been a shift in service location from the inpatient to the outpatient setting, where over two-thirds of TOP procedures in the US in 2005 were performed. Only 23% of TOP providers offer procedures after 20 weeks and only 11% after 24 weeks. TOP seems to be preferentially done in urban setting with 87% of US counties lacking a TOP provider [304]. 

 In response to a decline in the number of US OBG residents opting to not learn how to perform TOPs, and the subsequent decline in TOP providers, a privately-funded initiative was started in 1999 to try to reverse this trend. Over 40 US residencies have affiliated with the program. The program is named after Kenneth Ryan, who was the longstanding OBG Chair at Harvard and an advocate of legal TOP. Whether training is “routine” or “optional” remains a source of much discussion [305–307]. Federal funding of graduate medical education in the US provides residents with some protection on conscience and the freedom to choose whether to participate in TOP or not. It remains to be seen how the Affordable Care Act will impact conscience and resident decision-making about participation in TOP.

## 8. Law, Policy, and the Judiciary

 TOP is a contentious issue in US politics and culture and is a theme of both state and federal executive and legislative campaigns. Some of the contention is no doubt due to the fact that in 1973 the US Supreme Court in *Roe *versus* Wade* and *Doe *versus* Bolton* decreed an unrestricted right to TOP within the privacy provisions of the US Constitution. That decision overrode local restrictions in all states and territories and shifted the grounds for settling disputes on TOP from the legislative branch to the judiciary. Moreover, the decision overstepped cultural and religious prohibitions founded on the premise that fetus or embryos are at least potential individuals deserving moral status and societal protection [308].

 The 1973 decision did not find the right to TOP to be absolute, but did extend to that decision the highest degrees of constitutional protection, so-called strict scrutiny. Under the doctrine of strict scrutiny no state can restrict TOP unless there is a compelling state interest. The majority opinion found no state interest that would allow restrictions in the first trimester, only the health of the woman could be used to restrict TOP in the second trimester, and TOP in the third trimester (after viability) could be restricted except in situations where a woman's life or health was threatened. Health was broadly defined in the two decisions to include mental and emotional health. 

 Since 1973, the US Supreme Court has explicitly declined to overturn its decision on TOP. Despite that hesitancy, beginning in 1989 and with subsequent rulings, the Supreme Court has diminished the constitutional protection of the right for TOP and essentially degraded the standard of “strict scrutiny” that was originally assigned. The fruit of that change has been various state statutes requiring things such as a proscribed waiting period, mandatory informed consent, parental involvement for minors seeking TOP, and data reporting requirements. In 2007, the US Supreme Court went even further in *Gonzales *versus* Carhart* which upheld the “Partial Birth Abortion Ban Act of 2003.” That opinion allowed states to prohibit certain TOP techniques and for the first time there were no exceptions allowed for the health of the mother [309, 310]. 

 With the gradual relaxation of strict scrutiny, state legislatures and Congress began to regulate TOP in the US In 1976, the Hyde Amendment prohibited use of federal funds to pay for TOP procedures. States followed suit to forbid the use of state dollars (32 states and the District of Columbia) for TOP and to regulate insurance coverage for both public and private employees. Other restrictions include licensing and inspection requirements for TOP facilities, prohibition of certain procedures, mandatory information and waiting periods, required parental involvement, conscience clauses for both clinicians and institutions, and limitation of graduate medical education training requirements. Mandated ultrasounds prior to TOP with patients giving the option to view the images is the latest area where new state laws are trying to regulate TOP practices. TOP was a major issue in passage of the Affordable Care Act and how those laws will affect TOP funding and training for individuals and institutions await the Act's implementation [306]. Powerful public interest groups have risen on both sides of the restriction issue and are often on opposite sides of lawsuits to overturn new laws [310].

## 9. Conclusion

 The natural experiment in abolishing most restrictions on TOP initiated by the US Supreme Court in 1973 has proven the fallacies inherent in expert predictions. First, uptake of this procedure by women was far greater than predicted and in 2012 one out of three women in the US will have a TOP by the age of 45 [310, 311]. Second, a myopic focus on short-term complications has documented, albeit incompletely, the relative safety of the procedure at early gestational ages, but failed to adequately explore the long term health consequences. This is of particular importance with such widespread uptake of TOP where even modest increases in hazard ratios can have a huge impact on a population's health. The example of PTB stated herein is illustrative of this impact. The health impact of TOP is further befuddled by a paucity of reliable epidemiologic data on TOP and an inability to reliably know how often TOP is performed in the US and link administrative databases. Putting aside for a moment ethical considerations on the moral status of the embryo or fetus versus the status of his or her mother (which science is inadequate to address), there is a vacuum in TOP epidemiology and a real need to improve and upgrade our sources of observational data. Respect for maternal autonomy and the difficult decisions a woman faces in an unintended or crisis pregnancy must be considered cry out for nothing less. 
